# *Empetrum nigrum* var. *japonicum* Extract Suppresses Ultraviolet B-Induced Cell Damage via Absorption of Radiation and Inhibition of Oxidative Stress

**DOI:** 10.1155/2013/983609

**Published:** 2013-02-18

**Authors:** Ki Cheon Kim, Daeshin Kim, Sang Cheol Kim, Eunsun Jung, Deokhoon Park, Jin Won Hyun

**Affiliations:** ^1^School of Medicine and Institute for Nuclear Science and Technology, Jeju National University, Jeju 690-756, Republic of Korea; ^2^Halla Arboretum, Jeju 690-121, Republic of Korea; ^3^Biospectrum Life Science Institute, Seongnam 442-13, Republic of Korea

## Abstract

This study focused on the protective actions of *Empetrum nigrum* against ultraviolet B (UVB) radiation in human HaCaT keratinocytes. An ethyl acetate extract of *E. nigrum* (ENE) increased cell viability decreased by exposure to UVB rays. ENE also absorbed UVB radiation and scavenged UVB-induced intracellular reactive oxygen species (ROS) in HaCaT keratinocytes. In addition, ENE shielded HaCaT keratinocytes from damage to cellular components (e.g., peroxidation of lipids, modification of proteins, and breakage of DNA strands) following UVB irradiation. Furthermore, ENE protected against UVB-induced apoptotic cell death, as determined by a reduction in the numbers of apoptotic bodies and sub-G_1_ hypodiploid cells, as well as by the recovery of mitochondrial membrane potential. The results of the current study therefore suggest that ENE safeguards human keratinocytes against UVB-induced cellular damage via the absorption of UVB ray and scavenging of UVB-generated ROS.

## 1. Introduction

The ozone layer prevents ultraviolet B (UVB) radiation (wavelength range, 280–320 nm) from passing through the Earth's atmosphere [1]. However, depletion of the ozone layer has been observed in the stratosphere since the late 1970s, greatly increasing the risk of skin exposure to the detrimental actions of UVB radiation [2, 3]. UVB radiation directly damages DNA in skin cells such as keratinocytes due to the absorption of UVB photons by DNA [4]. UVB radiation also indirectly harms cellular components in irradiated skin tissues and cells via the generation of reactive oxygen species (ROS) from water, leading to oxidative DNA damage, lipid peroxidation, and protein modification [5].

Moderate UVB exposure is generally associated with the production of cutaneous vitamin D, which is essential for bone heath and the prevention of osteoporosis [6]. However, excessive UVB radiation is a contributory factor to the development of skin carcinoma, skin aging, and the suppression of skin immunity [7, 8]. Therefore, identification of protectants against UVB radiation has been the focus of intense study for decades. As human lifespan and cumulative sun exposure increase, the need to establish more powerful photoprotective compounds with limited adverse effects becomes more urgent. As such, research regarding a number of natural antioxidants has recently focused on the capacity to prevent or reduce UVB-mediated injury to the skin.

*Empetrum nigrum* var.* japonicum* is an evergreen plant that retains its leaves year round, even during winter and the dry season. *E. nigrum* is mainly found at high altitudes and in cold climates, such as hilly and mountainous areas. The plant bears fruit known as the black crowberry, which is consumed as a juice in Finland. Chalcone, dihydrochalcone, dihydrophenanthrene, and bibenzyl compounds have been isolated from *E. nigrum*, and consumption of *E. nigrum* fruit can reduce chronic human diseases as a result of the powerful antioxidant properties of these compounds [9–11]. 

Recently, our research has suggested that ENE can protect Chinese hamster lung fibroblasts (V79-4 cells) against hydrogen peroxide (H_2_O_2_-) induced cell damage via the scavenging of ROS and the enhancement of endogenous antioxidant enzyme activity [12]. Moreover, ENE prevented *γ*-ray-induced apoptosis in V79-4 cells via the inhibition of oxidative stress, as shown by restoration of antioxidant enzyme levels in the irradiated cells and attenuation of the c-Jun NH_2_-terminal kinase pathway [13]. Therefore, the present study evaluated the hypothesis that ENE can similarly shield human keratinocytes from UVB-induced oxidative stress and damage and explore its possible mechanism of action.

## 2. Materials and Methods

### 2.1. Preparation of ENE

The ethyl acetate fraction of *E. nigrum *(ENE) was kindly provided by Dr. Daeshin Kim (Halla Arboretum, Jeju, Republic of Korea). 

### 2.2. Cell Culture

Human keratinocytes (HaCaT cells) were obtained from the AmorePacific Corporation (Seoul, Republic of Korea) and cultured in Dulbecco's modified Eagle's medium containing 10% heat-inactivated fetal calf serum, streptomycin (100 *μ*g mL^−1^), and penicillin (100 U mL^−1^). Cells were maintained at 37°C in a humidified incubator containing 5% CO_2_ in air.

### 2.3. Reagents

[3-(4,5-Dimethylthiazol-2-yl)-2,5-diphenyltetrazolium] bromide (MTT), 2′,7′-dichlorodihydrofluorescein diacetate (DCF-DA), propidium iodide (PI), Hoechst 33342, and padimate O (2-ethylhexyl 4-dimethylaminobenzoate) were purchased from Sigma-Aldrich Co. (St. Louis, MO, USA). 5,5′,6,6′-Ttetrachloro-1,1′,3,3′-tetraethyl-benzimidazolcarbocyanine iodide (JC-1), and diphenyl-1-pyrenylphosphine (DPPP) were purchased from Molecular Probes (Eugene, OR, USA).

### 2.4. Cell Viability

The effect of ENE on the viability was determined using the MTT assay, which is based on the reduction of a tetrazolium salt by mitochondrial dehydrogenase in viable cells [14]. Cells were seeded in a 24-well culture plate at a density of 1 × 10^5^ cells mL^−1^ and treated with ENE (50 *μ*g mL^−1^) at 24 h after plating. One hour later, cells were exposed to UVB radiation at various radiation doses. A CL-1000 M UV Crosslinker (UVP, Upland, CA, USA) was used as the UVB source and delivered a UVB energy spectrum of 280–320 nm. Twenty-four hours later, MTT stock solution (100 *μ*L; 2 mg mL^−1^) was added to each well to yield a total reaction volume of 500 *μ*L. After incubating the cells with MTT for 2 h at 37°C, the supernatants were carefully aspirated with a suction pump. The formazan crystals in each well were dissolved in dimethylsulfoxide (DMSO; 500 *μ*L), and the absorbance at 540 nm was read on a scanning multiwell spectrophotometer.

### 2.5. UV/Visible Light Absorption Spectrum Analysis

An absorption analysis was performed for ENE (50 *μ*g mL^−1^) by scanning the extract with UV/visible light in the 250–500 nm range using an HP-8453E UV-visible spectroscopy system (Hewlett Packard, Palo Alto, CA, USA) and a standard quartz cuvette with a 1 cm path length. 

### 2.6. Detection of Intracellular ROS

HaCaT keratinocytes were treated with ENE (50 *μ*g mL^−1^) or padimate O 10 *μ*M and exposed to UVB radiation (150 mJ cm^−2^) 1 h later. Cells were incubated for an additional 24 h at 37°C. Following the addition of DCF-DA (25 *μ*M) [15], the fluorescence of the 2′,7′-dichlorofluorescein (DCF) product was detected using a PerkinElmer LS-5B spectrofluorometer (Becton, and Dickinson Company, Mountain View, CA, USA) and a FACSCalibur Flow Cytometer (Becton, and Dickinson Company). The fluorescence intensity in the cells was assessed from histograms generated with the CellQuest Pro software program (Becton, and Dickinson Company).

### 2.7. Nuclear Staining with Hoechst 33342

HaCaT keratinocytes were treated with ENE (50 *μ*g mL^−1^), exposed to UVB radiation (150 mJ cm^−2^) 1 h later, and incubated for an additional 24 h at 37°C. Hoechst 33342 (1.5 *μ*L; stock solution 10 mg mL^−1^), a DNA-specific fluorescent dye, was added to each well and incubated with the cells for 10 min at 37°C. The stained cells were visualized under a fluorescence microscope equipped with a CoolSNAP-Pro color digital camera (Meyer Instruments, Inc., Houston, TX, USA) to examine the degree of nuclear condensation.

### 2.8. Detection of Apoptotic Sub-G_1_ Hypodiploid Cells

PI flow cytometry was performed to determine the fraction of apoptotic sub-G_1_ cells with hypodiploid DNA content among HaCaT keratinocytes treated with ENE and/or UVB radiation, as previously described [16]. Keratinocytes were treated with ENE (50 *μ*g mL^−1^) for 1 h, then exposed to UVB radiation (150 mJ cm^−2^), and incubated for an additional 24 h at 37°C. Cells were then harvested and fixed in 70% ethanol (1 mL) for 30 min at 4°C. The cells were washed twice with phosphate buffered saline (PBS) containing 2 mM Ethylenediaminetetraacetic acid (EDTA) and incubated for 45 min in the dark at 37°C in PBS (1 mL) containing 200 *μ*g RNase A and 10 *μ*g PI. Flow cytometric analysis was performed by using a FACSCalibur flow cytometer (Becton, and Dickinson Company). The percentage of sub-G_1_ hypodiploid cells was assessed based on the histograms generated with the CellQuest Pro software program (Becton, and Dickinson Company).

### 2.9. Mitochondrial Membrane Potential (Δ*ψ*_*m*_) Analysis

HaCaT keratinocytes were treated with ENE (50 *μ*g mL^−1^) and exposed to UVB radiation (150 mJ cm^−2^) 1 h later. The cells were then incubated for another 12 h at 37°C. The Δ*ψ*_*m*_ was analyzed using JC-1, a lipophilic cationic fluorescent dye that enters mitochondria and changes from green to red with increasing membrane potential. JC-1 was added to each well, and cells were incubated for an additional 30 min at 37°C [17]. After washing with PBS, the stained cells were assayed using flow cytometry.

### 2.10. Lipid Peroxidation Assay

Lipid peroxidation was assessed by using DPPP as a probe [18]. DPPP reacts with lipid hydroperoxides to yield a fluorescent product, DPPP oxide, thus providing an indication of membrane damage. Cells were incubated with DPPP (5 *μ*M) for 15 min in the dark and were then treated with ENE (50 *μ*g mL^−1^) for 1 h before exposure to UVB radiation (150 mJ cm^−2^). Images of the DPPP fluorescence were captured at an excitation wavelength of 351 nm and an emission wavelength of 380 nm with a Zeiss Axiovert 200 inverted microscope equipped with a camera.

### 2.11. Protein Carbonyl Formation

Cells were treated with ENE (50 *μ*g mL^−1^) and exposed to UVB radiation (150 mJ cm^−2^) 1 h later. After incubation at 37°C for 24 h, the extent of protein carbonyl formation was assayed using an OxiSelect protein carbonyl ELISA kit from Cell Biolabs (San Diego, CA, USA) according to the manufacturer's instructions.

### 2.12. Single-Cell Gel Electrophoresis (Comet Assay)

An alkaline version of the comet assay was performed to determine the degree of oxidative DNA damage [19, 20]. A suspension of cells was mixed with 0.5% low melting temperature agarose (75 *μ*L; LMTA) at 37°C, and the mixture was spread on a fully frosted microscopic slide that was precoated with 1% normal melting temperature agarose (200 *μ*L). After the solidification of the agarose, the slide was covered with another 75 *μ*L of 0.5% LMTA and then immersed in a lysis solution (2.5 M NaCl, 100 mM NaEDTA, 10 mM Tris, 1% Trion X-100, and 10% DMSO, pH 10) for 1 h at 4°C. The slides were placed in a gel electrophoresis apparatus containing 300 mM NaOH and 10 mM NaEDTA (pH 13) for 40 min to allow for DNA unwinding and the expression of alkali-labile damage. Next, an electrical field was applied (300 mA, 25 V) for 20 min at 4°C to draw the negatively charged DNA toward the anode. After electrophoresis, the slides were washed three times for 5 min each time at 4°C in a neutralizing buffer (0.4 M Tris, pH 7.5), followed by staining with PI (75 *μ*L; 20 *μ*g mL^−1^). The slides were observed using a fluorescence microscope equipped with an image analyzer (Komet 5.5; Kinetic Imaging Ltd., Liverpool, UK). The percentage of the total cellular fluorescence in the DNA tails and the tail length of 50 cells per slide were recorded.

### 2.13. Detection of Dead Cells

Cells were treated with 0.25% trypsin/EDTA to obtain a single-cell suspension and then washed with PBS followed by a 30 min incubation with 20 *μ*g PI/mL to determine the percentage of dead cells by flow cytometry. Dead cells were quantified based on the dot plots generated by the BD CellQuest Pro software program (Becton, and Dickinson Company).

### 2.14. Statistical Analysis

All measurements were made in triplicate, and all values are expressed as the mean ± the standard error. The results were subjected to an analysis of variance (ANOVA) followed by the Tukey test to analyze differences between means. A *P* value of <0.05 was considered significant. 

## 3. Results

### 3.1. ENE Prevents UVB-Induced Cell Death

**Figure 1:**
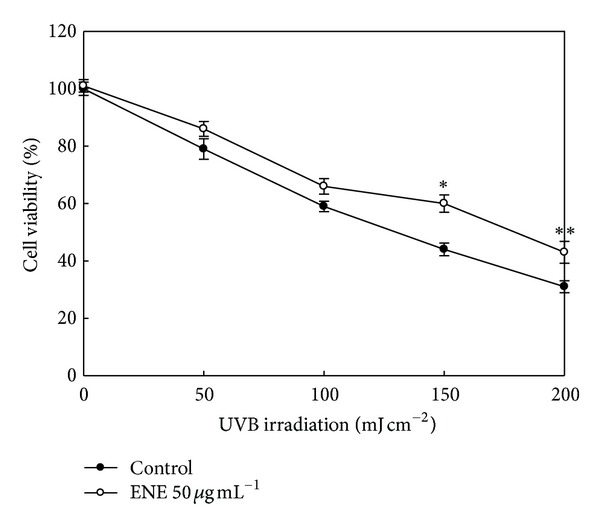
ENE restores cell viability in UVB-irradiated human HaCaT keratinocytes. Cells were pretreated with ENE (50 *μ*g mL^−1^) and exposed to UVB radiation 1 h later. Following an incubation period of 24 h, cell viability was assessed via the MTT assay. *Significantly different from UVB (150 mJ cm^−2^)-irradiated cells (*P* < 0.05); **significantly different from UVB (200 mJ cm^−2^)-irradiated cells (*P* < 0.05).

The ability of ENE to prevent cell death induced by UVB radiation was assessed by the MTT assay. As shown in Figure 1, cell viability in HaCaT keratinocytes treated with UVB radiation alone was decreased in a dose-dependent manner relative to cells that were not irradiated. Cell viability was approximately 100% in the absence of UVB radiation, whereas cell viability was reduced to 79% in cells treated with UVB at 50 mJ cm^−2^, 59% at 100 mJ cm^−2^, 44% at 150 mJ cm^−2^, and 31% at 200 mJ cm^−2^, respectively. Pretreatment of the UVB-irradiated cells with ENE (50 *μ*g mL^−1^) resulted in a significantly enhanced cell viability; cell viability was increased by 16% for cells pretreated with ENE, exposed to UVB at 150 mJ cm^−2^ and by 12% for cells pretreated with ENE, and exposed to UVB at 200 mJ cm^−2^. These results suggest that ENE protected human HaCaT keratinocytes from UVB-induced cell death.

### 3.2. ENE Absorbs UVB Light and Scavenges UVB-Generated ROS

**Figure 2:**
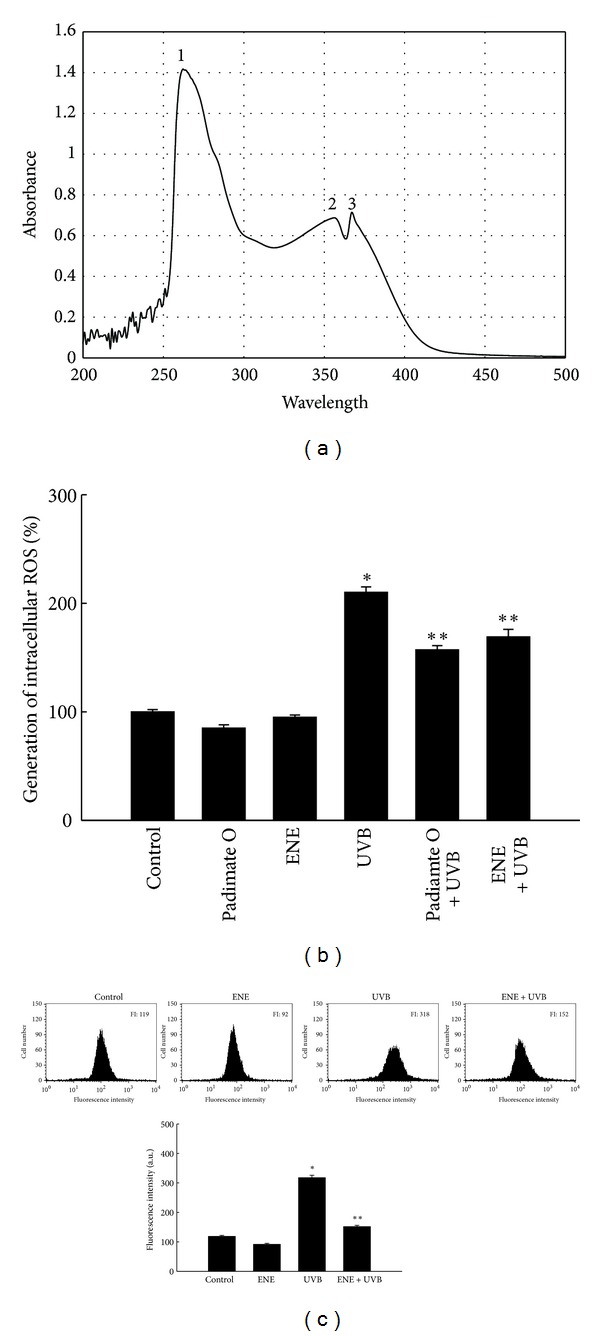
ENE absorbs UVB light and scavenges UVB-generated intracellular ROS in HaCaT keratinocytes. (a) The UV/visible spectroscopic measurement was conducted in the spectral range of 250–500 nm. Absorbance is reported in absorbance units. Peaks 1, 2, and 3 indicate peak positions at 262 nm, 356 nm, and 367 nm, respectively. (b) Intracellular ROS levels were measured using fluorescence spectrophotometry and (c) flow cytometry. *Significantly different from control cells (*P* < 0.05); **significantly different from UVB-irradiated cells (*P* < 0.05).

UVB radiation directly damages DNA due to the absorption of UVB photons by the DNA bases and indirectly damages cellular components (including DNA) via the generation of ROS [4, 5]. To elucidate the protective mechanism of ENE against UVB radiation, the ability of ENE to absorb UVB wavelengths and scavenge intracellular ROS in HaCaT keratinocytes was next assessed. ENE successfully absorbed electromagnetic radiation in the range of 280–320 nm, corresponding to the range of UVB radiation (Figure 2(a)). In addition, spectrofluorometry data showed that UVB-generated intracellular ROS levels increased to 210% from a control value of 100% in untreated cells. However, pretreatment of UVB-irradiated cells with UVB absorber padimate O and ENE resulted in a significant decrease to 157 and 169%, respectively (Figure 2(b)). These data are consistent with results from DCF flow cytometry; the ROS signal was 318 for UVB-irradiated cells versus 119, 92, and 152 for the untreated, ENE-only-treated and ENE-pretreated, and UVB-irradiated cells, respectively (Figure 2(c)).

### 3.3. ENE Ameliorates UVB-Induced Lipid Peroxidation, Protein Modification, and DNA Damage

**Figure 3:**
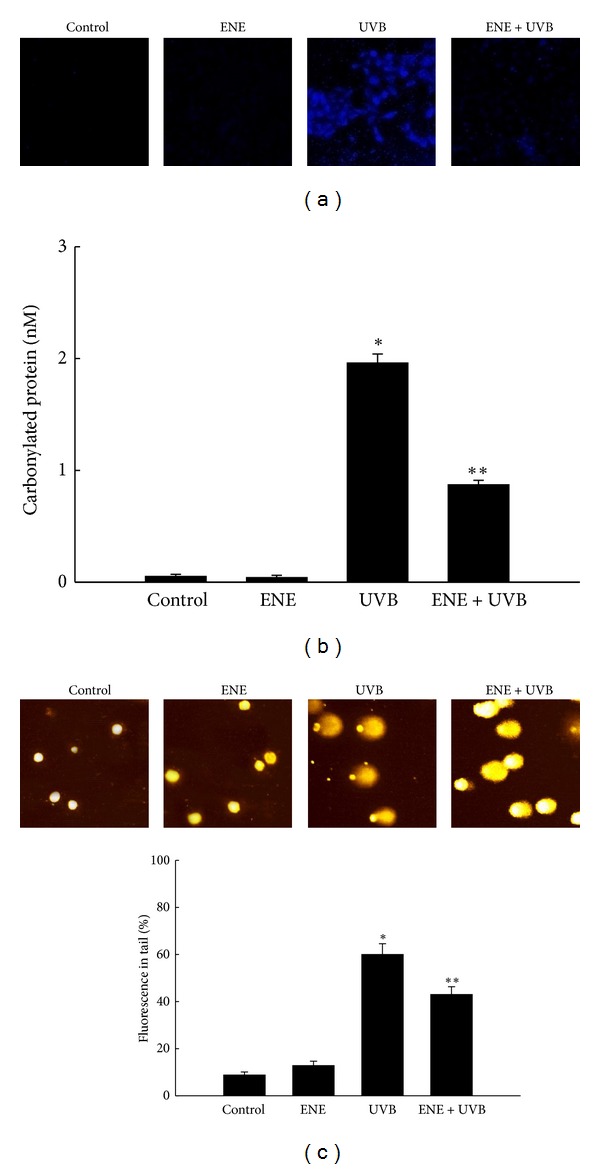
ENE protects HaCaT keratinocytes against UVB-induced lipid peroxidation, protein carbonylation, and DNA fragmentation. (a) Lipid hydroperoxide formation was detected by confocal microscopy after DPPP staining. (b) Protein oxidation was detected by measuring the amount of protein carbonyl formation. *Significantly different from control cells (*P* < 0.05); **significantly different from UVB-irradiated cells (*P* < 0.05). (c) DNA fragmentation was detected by an alkaline comet assay. *Significantly different from control cells (*P* < 0.05); **significantly different from UVB-irradiated cells (*P* < 0.05).

The ability of ENE to inhibit membrane lipid peroxidation, protein carbonyl formation, and DNA damage in UVB-irradiated cells was investigated. Lipid peroxidation was monitored by using DPPP, which specifically reacts with lipid hydroperoxides in cell membranes to yield the highly fluorescent product, DPPP oxide [18, 21]. The fluorescence intensity of DPPP oxide was enhanced in UVB-irradiated keratinocytes compared with untreated control cells. In contrast, pretreatment with ENE reduced the UVB-mediated increase in the DPPP oxide signal (Figure 3(a)). 

Oxidative stress stimulates the incorporation of carbonyl groups into the amino acid side chains of proteins [22]. UVB irradiation increased the carbonylated protein carbonyl content relative to untreated control. Treatment of the irradiated cells with ENE prevented the UVB-induced increase in protein carbonyl formation (Figure 3(b)). 

DNA fragmentation induced by UVB exposure was detected by an alkaline comet assay.  Figure 3(c) presents fluorescence microscopy images of the cellular nuclei and the percentage of cellular fluorescence in the comet tails for the untreated control, ENE-only treated, UVB-irradiated, and ENE-pretreated, UVB-irradiated cells. The exposure of cells to UVB compared with no treatment increased the tail length and the percentage of cellular fluorescence in the tails. The percentage of DNA in the tails increased to 60% for UVB-irradiated cells from 9% for untreated control cells. Pretreatment with ENE resulted in a significant decrease to 43% (Figure 3(c)). 

### 3.4. ENE Attenuates UVB-Induced Apoptotic Body Formation, Disruption of Δ*ψ*_*m*_ and Cell Death

**Figure 4:**
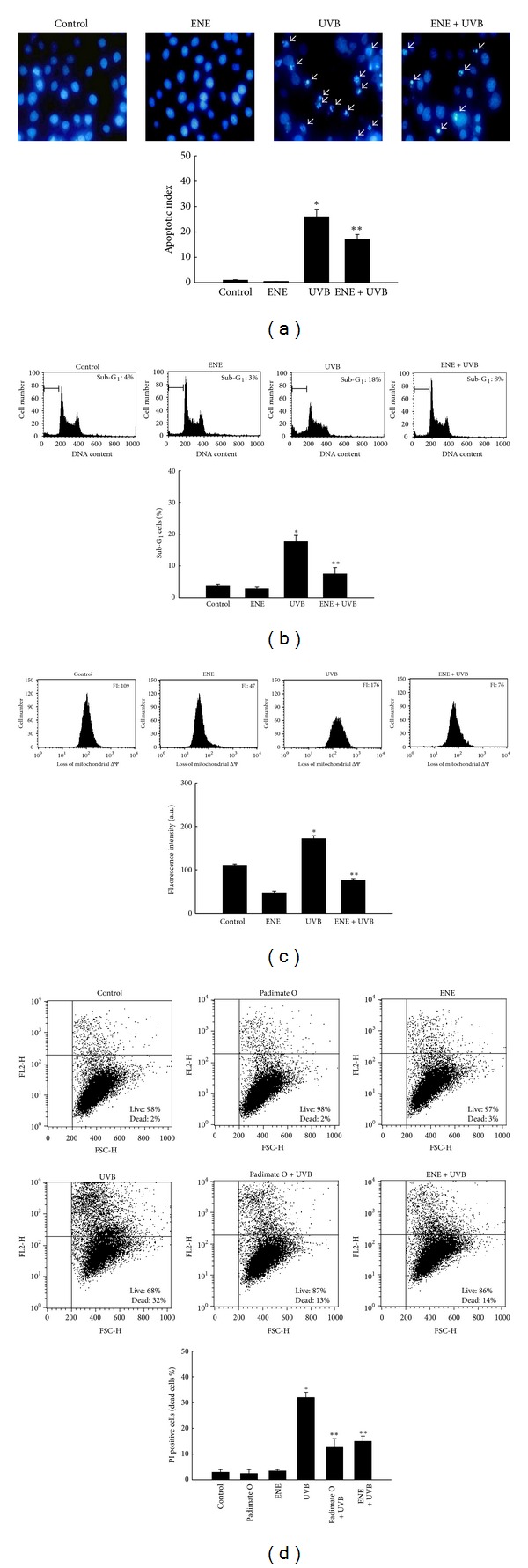
ENE protects HaCaT keratinocytes against UVB-induced apoptotic body formation and disruption of Δ*ψ*_*m*_. (a) Apoptotic bodies (arrows) were observed using fluorescence microscopy and quantified in cells stained with Hoechst 33342. The apoptotic index is defined as the number of apoptotic bodies divided by the total number of intact cells × 100. *Significantly different from control cells (*P* < 0.05); **significantly different from UVB-irradiated cells (*P* < 0.05). (b) Sub-G_1_ DNA content was measured by flow cytometry. *Significantly different from control cells (*P* < 0.05); **significantly different from UVB-irradiated cells (*P* < 0.05). (c) The Δ*ψ*_*m*_ was measured by flow cytometry after staining with JC-1. *Significantly different from control cells (*P* < 0.05); **significantly different from UVB-irradiated cells (*P* < 0.05). (d) PI-stained cells were measured using flow cytometry and the dead cell population was shown in the representative dot blot and histogram. *Significantly different from the control (*P* < 0.05); **significantly different from UVB- irradiated cells (*P* < 0.05).

UVB radiation induces apoptosis in keratinocytes, leading to cell damage and/or programmed cell death [23]. In Figure 4(a), the number of apoptotic bodies in the cellular nuclei divided by the total number of intact cells was employed as the apoptotic index. UVB-irradiated cells showed an apoptotic index of 26% versus 1% for untreated control; however, ENE pretreatment decreased this value to 17%. In addition, exposure to UVB radiation increased the percentage of cells with apoptotic sub-G_1_ hypodiploid DNA content to 18% from 4% for untreated control cells. ENE pretreatment decreased sub-G_1_ DNA content to 8% (Figure 4(b)). Finally, the Δ*ψ*_*m*_, a marker of mitochondrial membrane integrity, was detected using flow cytometry after staining of keratinocytes with the fluorescent dye JC-1. The flow cytometric data showed that UVB irradiation resulted in a loss of Δ*ψ*_*m*_, as determined by an increase in arbitrary fluorescence units from 109 for untreated control cells to 176 for UVB-treated cells. ENE treatment blocked the loss of Δ*ψ*_*m*_ in UVB irradiated-cells, as shown by a decrease in arbitrary fluorescence units to 76 (Figure 4(c)). Finally, cell death induced by UVB was quantified based on the PI staining of dead cells. In UVB-irradiated cells, 32% were PI positive compared with 3% of the control cells. In cells pretreated with padimate O and ENE prior to UVB irradiation, the percentage was 13 and 15%, respectively, which was significantly lower (Figure 4(d)).

## 4. Discussion

The stratospheric ozone layer absorbs solar UVB radiation and acts as a natural sunshield in the Earth's atmosphere. However, the ozone layer has been depleted over the past few decades due to the catalytic action of atomic halogens, such as chlorofluorocarbons, freons, and halons [2, 3]. As a consequence, human beings and other forms of life are at danger of overexposure to UVB radiation derived from the sun. Excessive exposure to UVB radiation leads directly to DNA damage and indirectly to the modification of cellular components via the generation of intracellular ROS [4, 5, 24–26]. UVB radiation also induces apoptosis, senescence, carcinogenesis, and mutagenic diseases in skin tissues and cells [6, 7, 27–30]. Although UVB radiation is not without its beneficial effects (e.g., stimulation of the synthesis of vitamin D, an essential component of the immune system that is associated with antitumor activity) [6, 31], the harmful effects of UVB rays on the skin are the subject of intensive UVB research. 

Since the initial reports regarding the thinning of the ozone layer, numerous studies have focused on bolstering skin protection against and resistance toward UVB radiation. Various natural products, such as green tea and blackberries, have been investigated both *in vivo* and *in vitro *as part of an arsenal for strengthening human skin against excessive UVB radiation [32–36]. Furthermore, many studies have indicated that the protective efficacy of compounds derived from these natural products stems from their antioxidant properties (specifically, scavenging of UVB-generated ROS) and their ability to absorb UVB radiation. For instance, myricetin, a phytochemical found in nuts and dark-pigmented fruits, significantly reduced tumor formation in mice that were chronically exposed to UVB radiation and quenched free radicals in the skin that were responsible for photoaging [37, 38]. Furthermore, most of the polyphenols originating from plants can absorb the entire spectrum of UVB wavelengths, as well as a partial spectra of UVC and UVA wavelengths [39]. As such, these polyphenols can block the incursion of UV photons into the skin and the subsequent damage that they cause to biomolecules. 

In the current study, ENE absorbed UVB wavelengths in the 280–320 nm range, which was likely due, at least in part, to its polyphenolic constituents. ENE also significantly reduced ROS generation in UVB-irradiated cells. Recently, we reported that ENE scavenged intracellular ROS induced by H_2_O_2_ and *γ*-radiation, as well as the hydroxyl radical and the superoxide anion [12, 13]. These results are consistent with the ROS scavenging activity of ENE demonstrated in this study. 

UVB radiation is a potent inducer of apoptosis that exerts its effects via damage to DNA and other cellular components, death receptor activation, and ROS generation [40, 41]. The predominant ROS generated by UVB include singlet oxygen, the superoxide anion, the hydroxyl radical, and H_2_O_2_. These ROS promote DNA, RNA, lipid, and protein oxidation. In this study, ENE protected cell membrane lipids and proteins in HaCaT keratinocytes from UVB-induced peroxidation damage, as demonstrated by decreased DPPP oxide fluorescence, and reduced levels of UVB-induced protein carbonyl formation. Moreover, ENE reduced DNA damage induced by UVB radiation, as observed by a reduction in the appearance of DNA tails in comet assays. In addition, ENE suppressed UVB-induced apoptotic cell death, as assessed by the decreased numbers of apoptotic bodies and a reduced Δ*ψ*_*m*_. 

In conclusion, the current study demonstrated that ENE safeguarded human HaCaT keratinocytes from UVB-induced cellular damage. The mechanisms of action of ENE against UVB radiation included (1) the direct absorption of UVB ray and (2) the scavenging of UVB-generated ROS. Therefore, ENE holds potential as a photoprotective agent for the shielding of skin from the detrimental effects of UVB exposure.
